# Clinical Manifestations, Prognostic Factors, and Outcomes of Extranodal Natural Killer T-Cell Lymphoma: A Single-Center Experience in Thailand

**DOI:** 10.3390/hematolrep16040073

**Published:** 2024-11-29

**Authors:** Wasinee Kaewboot, Lalita Norasetthada, Adisak Tantiworawit, Chatree Chai-Adisaksopha, Sasinee Hantrakool, Thanawat Rattanathammethee, Pokpong Piriyakhuntorn, Nonthakorn Hantrakun, Teerachat Punnachet, Ekarat Rattarittamrong

**Affiliations:** Department of Internal Medicine, Faculty of Medicine, Chiang-Mai University, Chiang-Mai 50200, Thailand; wasinee_kaew@cmu.ac.th (W.K.); lalita.n@cmu.ac.th (L.N.); adisak.tan@cmu.ac.th (A.T.); chatree.chai@cmu.ac.th (C.C.-A.); sasinee.h@cmu.ac.th (S.H.); thanawat.r@cmu.ac.th (T.R.); pokpong.p@cmu.ac.th (P.P.); nonthakorn.h@cmu.ac.th (N.H.); teerachat.pun@cmu.ac.th (T.P.)

**Keywords:** extranodal NK/T-cell lymphoma, prognostic index of natural killer lymphoma, Ann Arbor staging system, CA system

## Abstract

**Background/Objectives**: The primary objective of this study was to investigate clinical manifestations, time to diagnosis, and number of biopsies in patients with extranodal natural killer T-cell lymphoma (ENKTL). The secondary objectives were to determine response rates, survival outcomes, prognostic factor for overall survival (OS), and validation of the Prognostic Index of Natural Killer Lymphoma (PINK), Ann Arbor staging system (AASS), and the CA system. **Methods**: This retrospective study included data pertaining to patients with newly diagnosed ENKTL in Chiang-Mai University Hospital from 2004 to 2020. Comparisons between the areas under the receiver operating characteristic curve (AUC) of prognostic models (PINK, AASS, and CA system) were made. **Results**: Sixty patients were enrolled (n = 60) with a mean age of 49.1 ± 13.4 years. The most frequent symptom of ENKTL was nasal obstruction (66%). The median time to diagnosis was 22 days (ranging from 3 to 84 days), with 36.7% requiring more than one biopsy for diagnosis. Most patients presented with limited stage disease (75%). The median OS was 49 months. Factors associated with increased mortality were advanced stage, bone marrow involvement, gastrointestinal tract involvement, and receiving chemotherapy. Following prognostic model validation, the CA system model scored the highest level of accuracy (AUC 0.61), followed by AASS (AUC 0.58) and PINK (AUC 0.54). **Conclusions**: Patients with ENKTL commonly presented with nasal obstruction, with 36.7% requiring more than one biopsy for diagnosis. An advanced stage, bone marrow involvement, or gastrointestinal tract involvement were associated with poor OS. The CA system model has the highest level of accuracy for prognostic determination.

## 1. Introduction

Extranodal natural killer T-cell lymphoma (ENKTL) is a rare type of aggressive non-Hodgkin lymphoma (NHL) but is relatively common in Asia, accounting for 2.7% of all NHL in Thailand [1]. According to the Thai Lymphoma Study Group (TLSG) Registry, the median age of patients with ENKTL was found to be 46 years, with a male to female ratio of 2:1 [1]. ENKTL has been classified into nasal-type and extra-nasal-type, with an overall survival (OS) rate of 54% and 34% [2], respectively.

Patients with ENKTL mostly present with nasal symptoms [3], including nasal congestion, nasal discharge, nasal mass, epistaxis, or unspecific symptoms such as fever, headaches, or cervical lymph node enlargement. A diagnosis of ENKTL is established by histological and immunohistochemical examination [4]. ENKTL usually manifests as a necrotic lesion and angioinvasion [5], potentially requiring more than one diagnostic biopsy, which leads to a considerable delay in diagnosis. According to a study of 115 patients in China, 50 patients (43.5%), required more than one diagnostic biopsy to confirm a definitive diagnosis of ENKTL [3].

The modified Ann Arbor staging system (AASS) is mostly used for the staging of Hodgkin lymphoma and the majority of subtypes of NHL [6], which predominantly involves lymph nodes, potentially limiting its usefulness for the proper staging of ENKTL. The more recently developed prognostic models for staging or determining the prognosis of ENKTL include the Korean Prognostic Index (KPI) [7], Prognostic Index of Natural Killer Lymphoma (PINK) [8] and latterly, the Chinese Southwest Oncology Group and Asia Lymphoma Study Group ENKTL (CA system) [9]. A retrospective study comparing the CA system and the AASS in 205 patients found that the CA system had a more effective prognostic value than the AASS [10]. The KPI model has been acknowledged as having limitations since it was developed before the L-asparaginase-based therapy era [4]. However, there is no consensus regarding a standard prognostic model for the staging of ENKTL.

There is no standard treatment for patients with ENKTL. The use of a non-anthracycline-based regimen combined with radiotherapy (RT) is recommended for the treatment of limited-stage disease [4,11,12]. RT has an essential role in improving OS and progression-free survival (PFS) in patients with early-stage nasal ENKTL [13]. In the advanced stage, L-asparaginase-based chemotherapy is recommended [4,11,12].

Since there are limited clinical data and data relevant to the treatment outcomes of ENKTL in Thailand [1,14], the primary objective of this study was to determine the clinical manifestations, time to diagnosis, and number of biopsies in patients with ENKTL. The secondary objectives were to analyze response rates, OS, and prognostic factors for OS including validation of the PINK, modified AASS, and CA system.

## 2. Materials and Methods

This was a retrospective study conducted in Chiang Mai University Hospital from 1 January 2004 to 31 December 2020. We reviewed all data from patients aged > 18 years who were diagnosed with ENTKL according to the World Health Organization (WHO) classification [15] through medical records. In cases where Epstein–Barr virus-encoded RNA (EBER) status was not available, the diagnosis was based on WHO classification at the time of diagnosis (third, fourth, or revised fourth edition). All patients had a biopsy-proven diagnosis with histopathological and immunohistochemical studies to confirm the diagnosis of ENKTL by hematopathologists and had clinical data including age, sex, clinical presentation, site of presentation, performance status, time to diagnosis, number of biopsies, laboratory parameter at diagnosis, imaging, treatment modalities, response outcomes according to Lugano Classification [6] (complete response [CR], partial response [PR], stable disease [SD], and progressive disease [PD]), survival outcomes, and follow-up for at least 5 years. This study was approved by the Institutional Research Committee of the Faculty of Medicine, Chiang Mai University, and was conducted in accordance with the Declaration of Helsinki (Certificate of Ethical Approval no. 185/2022).

### Statistical Analysis

We analyzed the data pertinent to clinical presentations, laboratory, histology, stage, and treatment, with data being presented as descriptive analyses. All dichotomous variables are presented as percentage or ratio. All continuous variables are presented as mean ± standard deviation or medians and interquartile ranges (IQRs) as appropriate. Factors affecting OS and PFS were analyzed by univariate and multivariate methods using the Cox proportional hazard model. Survival data were analyzed using the Kaplan–Meier method. Receiver operating characteristic (ROC) curves were generated, and the area under the curve (AUC) was calculated to assess the predictive ability of the AASS, PINK, and the CA systems in predicting overall survival. Statistical significance was set at *p* < 0.05 (two-sided). STATA version 17.0 was used for data analysis. The sample size calculation was based on a similar previous study [7]. The CR rate was 56%, and the hazard ratio that we wanted to detect was set at 2.5–3. With this evidence, a minimum of 66 to 95 patients were required to achieve 80% power and a two-sided type I error of 5%.

## 3. Results

### 3.1. Patient Characteristics

During the 16-year eligibility window, a total of 60 patients diagnosed with ENKTL were eligible for inclusion in this study. The clinical characteristics and demographic data are shown in Table 1. The median age was 49 years (range 36–62 years), with 39 of the patients (65%) being male. Forty-five patients (75%) were in the limited stage of the disease (modified AASS I-II), while 15 patients were in the advanced stage (modified AASS III–IV). Most patients (86.7%) presented with nasal symptoms, including nasal obstruction (66.7%), followed by purulent nasal discharge (45%), bloody nasal discharge (16.7%), nasal mass (26.7%), and anosmia (11.7%). Thirty-seven patients (61.7%) had B symptoms. The primary sites were the nasal cavity (68.3%), paranasal sinus (25%), nasopharynx (11.7%), and oropharynx (11.7%). Thirty-eight patients (63.3%) underwent one biopsy for the diagnosis of ENKTL, while twenty-two patients (36.7%) required more than one biopsy. Median time to diagnosis was 22 days (range from 3 to 84 days). Three patients (5%) had bone marrow involvement, while two patients (3%) had gastrointestinal involvement. Epstein–Barr virus-encoded RNA (EBER) was positive in 15 out of 20 patients (75%).

### 3.2. Treatment and Response Outcomes

#### 3.2.1. Limited Stage

As shown in Table 2, about 60% of the patients received concurrent chemoradiotherapy (CCRT) (29 patients, 64.4%) followed by chemotherapy alone (6 patients, 13.3%), RT then chemotherapy (4 patients, 8.9%), RT alone (3 patients, 6.7%), and chemotherapy then RT (3 patients, 6.7%). A variety of chemotherapy regimens were used, as shown in Table 3. Most patients were given anthracycline-containing regimens and RT. The overall response rate (ORR) was 77.8% (35 patients). Relapsed disease occurred in 13 patients (37.1%).

#### 3.2.2. Advanced Stage

The majority of the patients (11 patients, 73.3%) received chemotherapy alone followed by CCRT (3 patients, 20%) and chemotherapy then RT (1 patient, 6.7%). Response was achieved in about half of patients (8 patients, ORR 53.3%) and all had relapsed disease (8 patients, 100%).

### 3.3. Overall Survival

**Figure 1:**
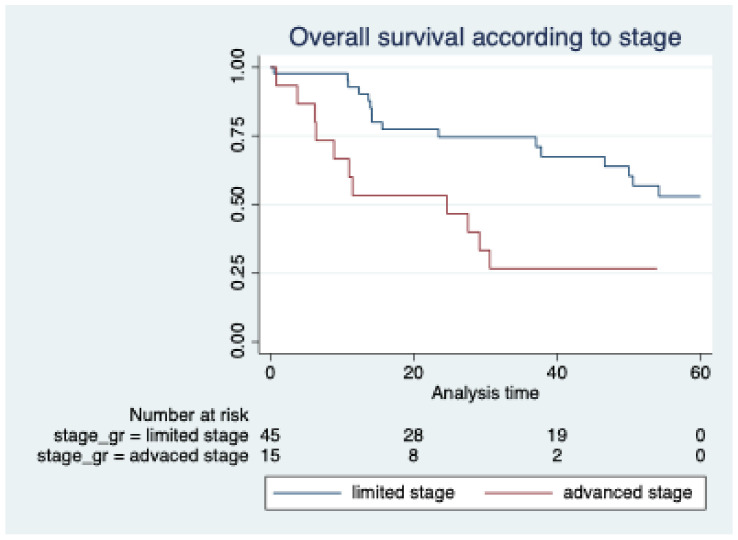
Kaplan–Meier curves showing overall survival according to the stage of disease.

The median follow-up time was 60 months. Median OS was 49 months. The median OS in limited stage disease was not reached, and that in advanced stage disease was 24.5 months (95% CI 6–30 months), which was significantly different (*p* = 0.002) (Figure 1). The cause of death was mostly infection (9 patients, 33.3%), followed by progression of disease (1 patient, 3.7%) and complications of chemotherapy (1 patient, 3.7%), while the cause was unknown in the remaining cases (16 patients, 59%).

**Figure 2:**
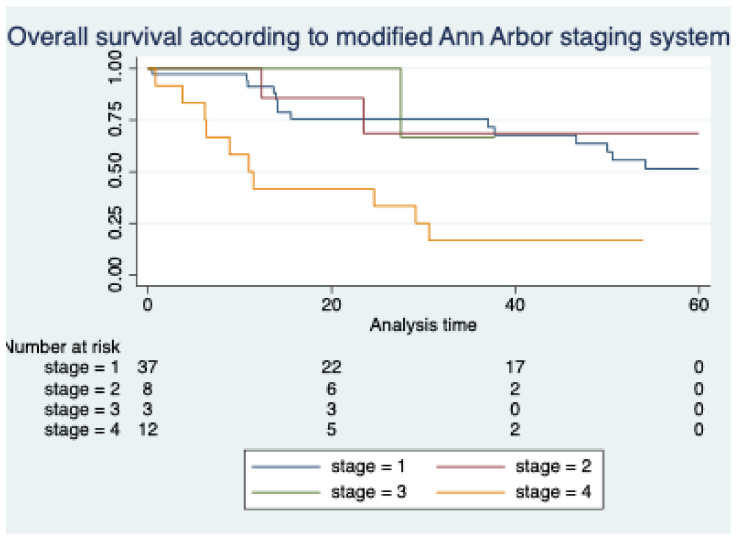
Kaplan–Meier curves showing overall survival according to modified AASS.

According to the modified AASS, the distribution of staging from stage I through IV was 61.6%, 13.3%, 5% and 20%, respectively. The 5-year OS rates for modified AASS indicate that the OS of patients with stage IV was worse, while there was no difference between stages I through III (10 months vs. not reached, *p* = 0.0018) (Figure 2).

**Figure 3:**
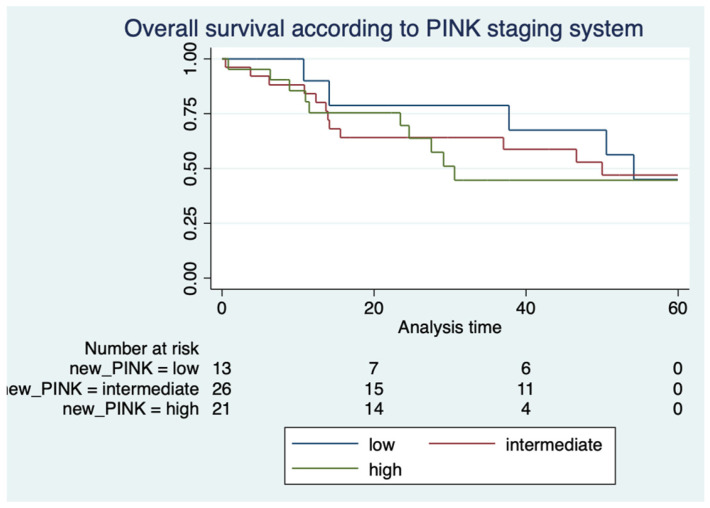
Kaplan–Meier curves showing overall survival according to PINK.

Following application of the PINK prognostic score, 21.7%, 43.3%, and 35% of patients were categorized as low, intermediate, and high risk, respectively. The median OS in patients categorized by PINK was 54 months, 50 months, and 30 months, respectively (*p* = 0.79) (Figure 3).

**Figure 4:**
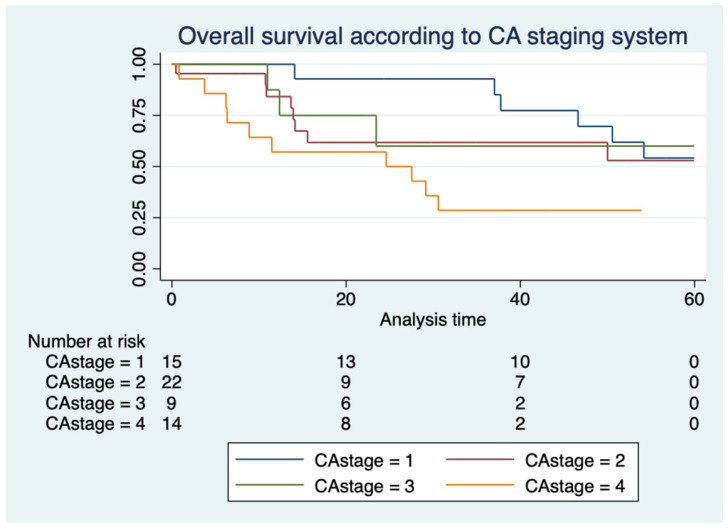
Kaplan–Meier curves showing overall survival according to CA staging system.

The distribution of staging according to the CA system from stage I to stage IV was 25%, 36.7%, 15% and 23.3%, respectively. The median OS of stage I through III was not reached, while stage IV had a median OS of 24 months (*p* = 0.059) (Figure 4).

**Figure 5:**
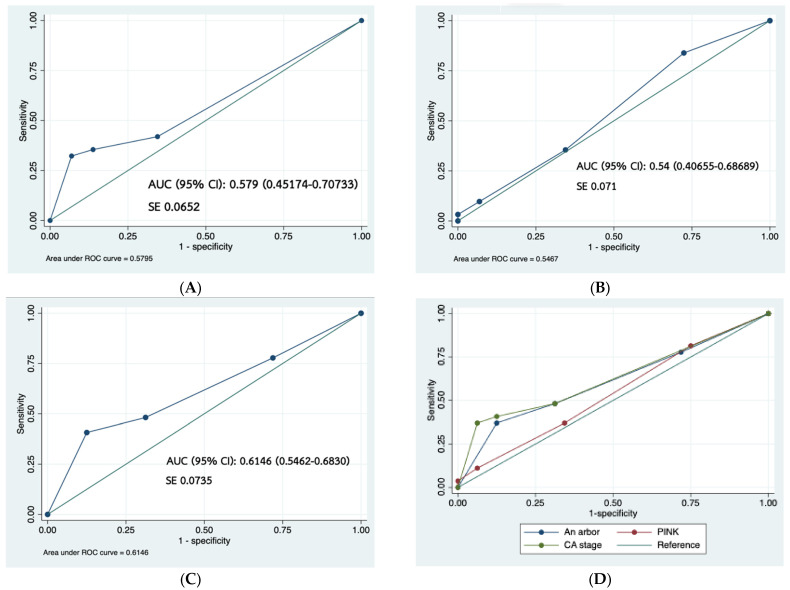
ROC analysis of modified AASS ((**A**): Blue line), PINK ((**B**): Blue line), CA system ((**C**): Blue line), and all models (**D**). The green lines in subfigure (**A**–**C**) represent reference.

In the ROC analysis, the area under the curve (AUC) of modified AASS was 0.58, PINK was 0.54, and the CA system was 0.61 (Figure 5); therefore, the CA system could be said to more accurately discriminate for survival than the modified AASS and PINK.

**Figure 6:**
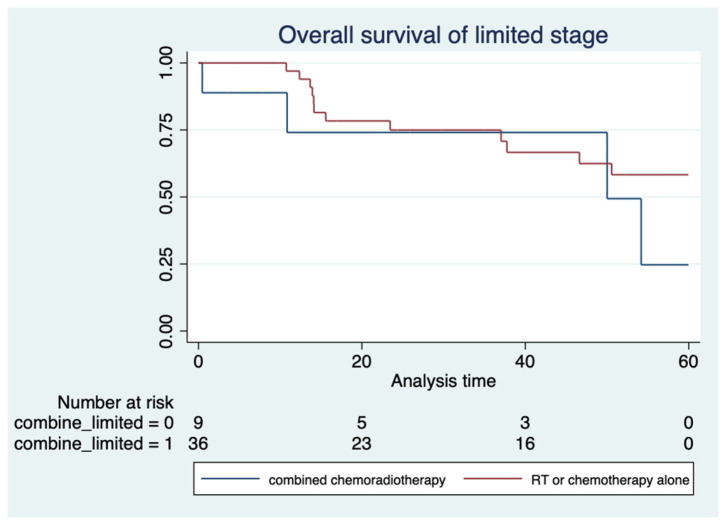
Overall survival of patients with limited stage disease treated with combined chemoradiotherapy (red line) and chemotherapy or radiotherapy alone (blue line).

In limited stage disease, the median OS of the combined chemoradiotherapy group was not reached, while that following RT or chemotherapy alone was 50 months (*p* = 0.269) (Figure 6). The most common chemotherapy regimens used were CHOP, CHOP-L-asparaginase, AspaMetDex, BMAD (clinical trial from the TLSG), GELOX, and SMILE. (Table 3)

**Figure 7:**
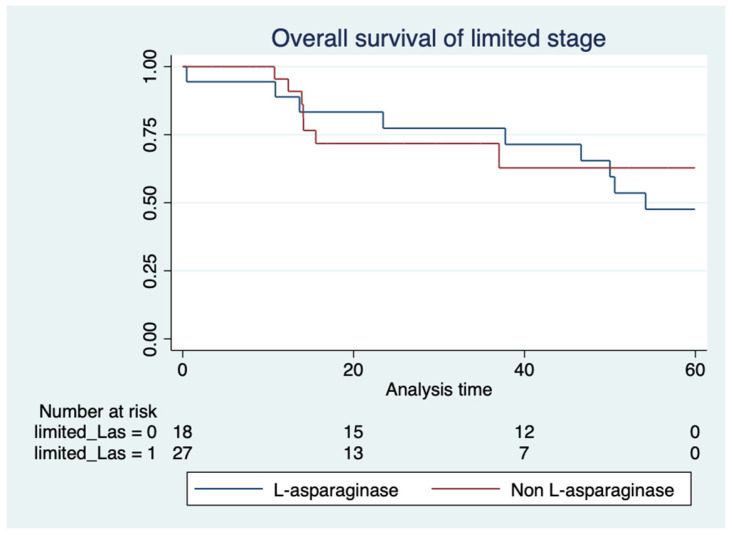
Overall survival of patients with limited stage disease according to L-asparaginase-based regimen.

**Figure 8:**
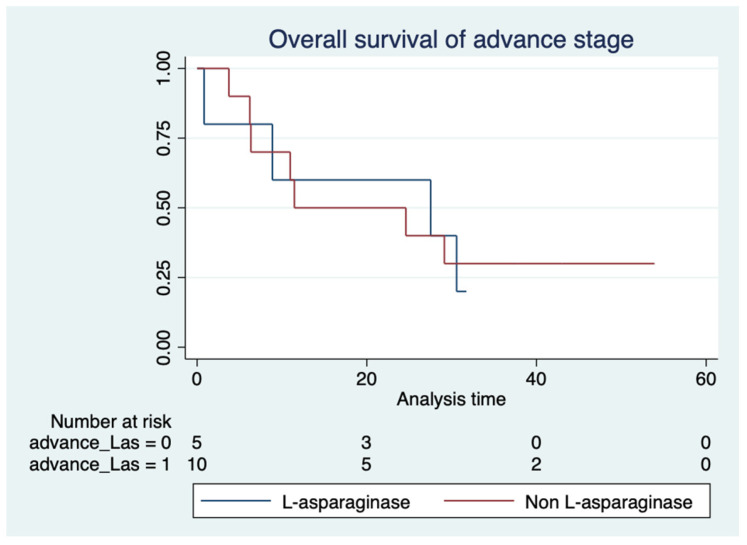
Overall survival of patients with advanced stage disease according to L-asparaginase-based regimen.

Regarding chemotherapy, the L-asparaginase-based regimen did not show significant differences in OS compared with a non-L-asparaginase-based regimen for either limited stage (median OS not reached vs. 54.1 months, *p* = 0.69) (Figure 7) or advanced stage disease (median OS 27.5 vs. 11.4 months, *p* = 0.89) (Figure 8).

The results of the Cox univariate and multivariate proportional hazard analysis showed that the factors associated with increased mortality among all newly diagnosed ENKTL patients were advanced stage (unadjusted HR 3.28, 95% CI 1.48 to 7.28, *p* = 0.004), bone marrow involvement (unadjusted HR 7.14, 95% CI 2.05 to 24.90, *p* = 0.002), gastrointestinal tract involvement (adjusted HR 12.21, 95% CI 1.75–85.33, *p* = 0.012), and receiving chemotherapy (alone or in combination with RT compared to RT alone) (adjusted HR 5.02, 95% CI 1.65–15.26, *p* = 0.005) (Table 4). The number of biopsies (adjusted HR 2.51, 95% CI 0.73–8.65, *p* = 0.145) and time to diagnose (adjusted HR 2.51, 95% CI 0.73–8.65, *p* = 0.145) were not significantly associated with OS.

## 4. Discussion

This retrospective study investigated clinical presentations, time to diagnosis, number of biopsies, prognostic factors, and treatment outcomes of patients with ENKTL. Epidemiology data were comparable with other studies from Thailand [1,14] and Asian countries [3,9,16], including a proportion of patients aged less than 60 of around 70–80%, a slight male predominance, a rate of nasal-type presentation of approximately 80%, and a majority of patients in the limited stage. The most common primary site was the nasal cavity (68.3%). The most common clinical presentations were nasal obstruction (66.7%) followed by purulent nasal discharge, bloody nasal discharge, nasal mass, and anosmia. The finding that nasal obstruction or congestion were the most common symptoms was similar to other case series from China (73.0%) [3] and India (66.7%) [17].

ENKTL usually manifests as a necrotic lesion and angioinvasion, frequently requiring more than one diagnostic biopsy, therefore leading to a considerable delay in diagnosis. In our study, 38 patients (63.3%) underwent only one biopsy procedure to make a correct diagnosis of ENKTL, but two or more biopsies were needed in 22 patients (36.7%). This finding corresponded with a previous study from China [3] which reported that two or more biopsies were needed in 50 patients (45.5%) to reach the correct diagnosis. The median time to diagnosis in our study was 22 days (range from 3 to 84 days), while a previous study in North America [18] reported a median time to diagnosis of 5 months (range 1–36). This might be due to a low level of suspicion of ENKTL in the US and its incorrect interpretation as an invasive bacterial or fungal sinusitis. As a result, multiple biopsies should be considered for confirmation when ENKTL is clinically suspected. EBER staining in tissue biopsy was performed in a quarter of patients after the test became available. The result showed positive EBER in 75% of patients, which is less than expected (100%) [14,16]. This might have been due to extensive necrosis of tissue, affecting reliable evaluation.

The analysis of treatment outcomes did not show a better median OS in patients treated with combined chemoradiotherapy than in those treated with chemotherapy or RT alone in limited stage ENKTL (*p* = 0.269). The findings are in contrast with the report by the international T-cell project that showed definite survival benefits associated with chemoradiation (3-year OS, 70%), which was more effective than chemotherapy alone (3-year OS, 12%) in patients with localized ENKTL [2].

It should be noted that 40% of the patients in limited stage disease and one-third in advanced stage disease in this study did not receive an L-asparaginase-based regimen but received anthracycline-based chemotherapy. This group of patients were treated during 2004–2010, which was the period before the widespread adoption of L-asparaginase in frontline regimens. However, this study did not discover any significant benefit of the L-asparaginase-based regimen in either the limited stage (median OS not reached vs. 54.1 months, *p* = 0.69) or advanced stage disease of ENKTL (median OS 27.5 vs. 11.4 months, *p* = 0.89). This finding was discordant from a previous study in China [19], which compared the efficacy of an L-asparaginase-based regimen and CHOP regimen followed by RT as first-line treatments for newly diagnosed ENKTL. That study demonstrated that the 5-year OS of the L-asparaginase group was 65.1%, compared to 25.8% in the CHOP group (*p* < 0.001). Another study from Thailand also demonstrated non-significant improvement in PFS (median PFS 32 months vs. 3 months, *p* = 0.06) and OS (median OS 33 months vs. 11 months, *p* = 0.22) in ENKTL patients who received an L-asparaginase-based regimen [18]. This discordant result might be explained by different chemotherapy regimens and varied populations. In addition, our study did not aim to compare different chemotherapy regimens, so this result should be interpreted with caution due to the extremely small number of patients analyzed and may be subject to bias due to the underpowered retrospective and single-institution nature of this study.

Our study revealed that using the modified AASS, the distribution of patients from stage I through IV was 61.6%, 13.3%, 5%, and 20%, respectively. However, according to the CA system, the distribution was 25%, 36.3%, 15%, and 23%, respectively. ENKTL is mostly an extra-nodal lymphoma with localized lesions; hence use of the AASS might be not suitable for indicating the extent of local tumor invasion. The CA system was found to have a better prognostic value than the modified AASS (AUC of ROC analysis 0.61 vs. 0.58). This finding corresponds with a previous study in China [10], which showed an AUC of 0.70 for the CA system and 0.64 for the AASS. The high-risk patients determined by the CA system should be considered for enrollment in clinical trials [9]. In the case of the PINK model, the AUC is very similar to that of the AASS. However, we did not validate PINK-E [8] in this study because assessment of the EBV viral load was not routinely performed in our center. Various novel prognostic markers for patients with ENKTL were investigated including pre-treatment serum C-reactive protein [20], controlling nutritional status (CONUT) score [21], 25-hydroxy vitamin D [22], the maximum standardized uptake value (SUVmax) from ^18^F-fluorodeoxyglucose positron emission tomography/computed tomography (^18^F-FDG PET/CT) [23], and circulating tumor DNA [24]; these, as well as novel prognostic models [22,23,24,25] should be validated further.

Some limitations of this study warrant discussion. Firstly, we cannot exclude selection bias and confounding variables that occur due to the nature of any retrospective study. Secondly, incomplete data collection may have affected the accuracy of our study. Thirdly, the small sample size and single-center analysis may limit the generalizability of our results; consequently, the analysis of outcomes may not be accurately interpreted.

## 5. Conclusions

Nasal symptoms were the most common presentation of ENKTL, and the nasal cavity was identified as the most common primary site in patients with ENKTL. Nearly 40% of the patients required more than one diagnostic biopsy. The CA system demonstrated more accurate discrimination with regard to overall survival than the modified AASS and PINK. The validation of these prognostic scores in larger and different populations is warranted.

## Figures and Tables

**Table 1:** Clinical characteristics of extranodal NK/T-cell lymphoma patients.

Characteristic	Total No. (%) n = 60	Limited Stage No. (%) n = 45	Advance Stage No. (%) n = 15	*p* Value
Age, years (mean ± SD)	49.1 ± 13.4			
≤60	47 (78.33)	35 (77.78)	12 (80.00)	0.856
>60	13 (21.67)	10 (22.22)	3 (20.00)	
Male	39 (65)	32 (71)	7 (46.7)	0.086
Nasal symptoms	52 (86.7)	41 (91.1)	11 (73.3)	0.098
Nasal obstruction	40 (66.7)	33 (73.3)	7 (46.7)	0.058
Purulent nasal discharge	27 (45)	21 (46.7)	6 (40)	0.653
Bloody nasal discharge	10 (16.7)	9 (20)	1 (6.7)	0.426
Nasal mass	16 (26.7)	11 (24.4)	5 (33.3)	0.500
Anosmia	7 (11.7)	6 (13.3)	1 (6.7)	0.486
Extranasal symptoms	27 (45)	21 (46.7)	6 (40)	0.653
B symptoms	37 (61.7)	28 (62.2)	9 (60)	0.878
Primary site				
Nasal cavity	41 (68.3)	31 (68.9)	10 (66.7)	0.873
Paranasal sinus	15 (25)	11 (24.4)	4 (26.7)	0.863
Nasopharynx	7 (11.7)	7 (100)	0 (0)	0.176
Oropharynx	7 (11.7)	6 (13.3)	1 (6.67)	0.668
Other	17 (28.3)	10 (22.2)	7 (46.7)	0.069
Number of biopsies (n)				
<2	38 (63.3)	29 (76.3)	9 (23.7)	0.757
≥2	22 (36.67)	16 (72.73)	6 (27.27)	
Times to diagnosis, months				
0–1 month	44 (73.33)	33 (73.33)	11 (73.33)	0.646
2–3 months	11 (18.33)	9 (20.00)	2 (13.33)	
>3 months	5 (8.33)	3 (6.67)	2 (13.33)	
ECOG performance status				
0–1	57 (95)	43 (95.5)	14 (95.3)	0.184
≥2	3 (5)	2 (4.5)	1 (6.67)	
Bone marrow involvement	3 (5)	0 (0)	3 (20.0)	0.013
GI involvement	2 (3.33)	0 (0)	2 (13.3)	0.059
Increased serum LDH	14 (23.73)	10 (22.73)	4 (26.67)	0.757
Serum albumin ≤ 35 g/L	18 (30)	14 (31.1)	4 (26.7)	0.745
ALC ≤ 1000 per mm^3^	11 (18.3)	8 (17.8)	3 (20.0)	0.847
Hb ≤ 100 g/L	11 (18.3)	9 (20.0)	2 (13.3)	0.714
EBER detected	15 (75.00)	12 (80.0)	3 (60.0)	0.371
Median follow-up time, months	23.7	27.4	12.7	-

Abbreviations: LDH: Lactate dehydrogenase; ALC: absolute lymphocyte count; Hb: Hemoglobin; EBER: Epstein–Barr virus-encoded RNA.

**Table 2:** Treatment and response outcomes.

Treatment	Limited Stage (N = 45)			Advanced Stage (N = 15)		
	N (%)	Overall Response (%)	Relapse (%)	N (%)	Overall Response (%)	Relapse (%)
Radiotherapy alone	3 (6.7)	3 (100.0)	0 (0)	0 (0)	-	-
Chemotherapy alone	6 (13.3)	3 (50.0)	2 (66.7)	11 (73.3)	4 (36.4)	4 (100)
Chemotherapy then radiotherapy	3 (6.7)	3 (75.00)	1 (33.3)	1 (6.7)	1 (100)	1 (100)
Concurrent chemoradiotherapy	29 (64.4)	23 (79.3)	9 (39.1)	3 (20.0)	3 (100)	3 (100)
Radiotherapy then chemotherapy	4 (8.9)	3 (75.0)	1 (33.3)	0 (0)	-	-

**Table 3:** Chemotherapy regimens and treatment response.

Chemotherapy Regimen	n	Limited Stage			n	Advanced Stage		
		CR (%)	PR (%)	Relapse (%)		CR (%)	PR (%)	Relapse (%)
CHOP	15	13 (72.2)	2 (11.1)	6 (40)	4	2 (40)	0 (0)	2 (100)
CHOP + Lasp	8	6 (75.0)	0 (0.00)	3 (50)	3	1 (33.3)	0 (0.00)	1 (100)
AspaMetDex	3	1 (33.3)	1 (33.3)	0 (0)	6	2 (33.3)	0 (0.00)	2 (100)
BMAD	7	5 (71.4)	0 (0.00)	2 (40)	2	0 (0.00)	2 (100)	2 (100)
GELOX	8	5 (62.5)	0 (0.00)	2 (40)	0	0 (0)	0 (0)	0 (0)
SMILE	1	0 (0)	0 (0)	0 (0)	0	0 (0)	0 (0)	0 (0)

CHOP: cyclophosphamide, doxorubicin, vincristine, prednisolone; Lasp: L-asparaginase; AspaMetDex: L-asparaginase, methotrexate, dexamethasone; BMAD: brentuximab vedotin, methotrexate, L-asparaginase, dexamethasone; GELOX: gemcitabine, oxaliplatin, L-asparaginase; SMILE: dexamethasone, methotrexate, ifosfamide, L-asparaginase, etoposide.

**Table 4:** Univariate and multivariable hazard analysis for overall survival.

Factors	Overall Survival			
	Univariate Model		Multivariable Model	
	Unadjusted HR (95% CI)	*p*-Value	Adjusted HR (95% CI)	*p*-Value
Stage (advanced vs. limited)	3.28 (1.48–7.28)	0.004	1.02 (0.33–3.13)	0.975
Bone marrow involvement	7.14 (2.05–24.90)	0.002	3.15 (0.75–13.21)	0.117
Gastrointestinal involvement	24.09 (3.95–147.16)	0.001	12.21 (1.75–85.33)	0.012
Chemotherapy	5.32 (2.46–11.53)	<0.001	5.02 (1.65–15.26)	0.005

## Data Availability

The data that support the findings of this study are available from the corresponding author [E.R.], upon reasonable request.
